# Solid-phase synthesis of d-fructose-derived Heyns peptides utilizing N^α^-Fmoc-Lysin[N^ε^-(2-deoxy-d-glucos-2-yl),N^ε^-Boc]-OH as building block

**DOI:** 10.1007/s00726-021-02989-7

**Published:** 2021-05-02

**Authors:** Sebastian Schmutzler, Daniel Knappe, Andreas Marx, Ralf Hoffmann

**Affiliations:** 1 Institut Für Bioanalytische Chemie, Fakultät Für Chemie Und Mineralogie, Universität Leipzig, Leipzig, Germany; 2 Biotechnologisch-Biomedizinisches Zentrum, Universität Leipzig, Deutscher Platz 5, 04103 Leipzig, Germany; 3 Site Management - Analytics, Merck KGaA, Darmstadt, Germany

**Keywords:** Fructose, Glycation, Heyns compound, Maillard reaction, N^ε^-glucosyllysin building block, Solid-phase peptide synthesis (SPPS)

## Abstract

Aldoses and ketoses can glycate proteins yielding isomeric Amadori and Heyns products, respectively. Evidently, d-fructose is more involved in glycoxidation than d-glucose favoring the formation of advanced glycation endproducts (AGEs). While Amadori products and glucation have been studied extensively, the in vivo effects of fructation are largely unknown. The characterization of isomeric Amadori and Heyns peptides requires sufficient quantities of pure peptides. Thus, the glycated building block N^α^-Fmoc-Lys[N^ε^-(2-deoxy-d-glucos-2-yl),N^ε^-Boc]-OH (Fmoc-Lys(Glc,Boc)-OH), which was synthesized in two steps starting from unprotected d-fructose and Fmoc-l-lysine hydrochloride, was site-specifically incorporated during solid-phase peptide synthesis. The building block allowed the synthesis of a peptide identified in tryptic digests of human serum albumin containing the reported glycation site at Lys233. The structure of the glycated amino acid derivatives and the peptide was confirmed by mass spectrometry and NMR spectroscopy. Importantly, the unprotected sugar moiety showed neither notable epimerization nor undesired side reactions during peptide elongation, allowing the incorporation of epimerically pure glucosyllysine. Upon acidic treatment, the building block as well as the resin-bound peptide formed one major byproduct due to incomplete Boc-deprotection, which was well separated by reversed-phase chromatography. Expectedly, the tandem mass spectra of the fructated amino acid and peptide were dominated by signals indicating neutral losses of 18, 36, 54, 84 and 96 *m/z*-units generating pyrylium and furylium ions.

**Supplementary Information:**

The online version contains supplementary material available at 10.1007/s00726-021-02989-7.

## Introduction

Protein glycation is a non-enzymatic posttranslational modification that was linked to diabetes, rheumatoid arthritis, Alzheimer’s disease, and inflammatory diseases. Glycation is the initial stage of the Maillard reaction characterized by the reaction of electrophilic carbonyl groups, e.g., reducing sugars, with nucleophilic amino groups to form a Schiff base, which can rearrange to glycation products. Aldoses, such as d-glucose as dominant sugar in blood, can react with the ε-amino groups of lysine residues yielding 1-deoxy-1-ketosyllysine, i.e., fructosyllysine (glucated lysine), also referred to as Amadori product. In contrast, the rearrangement induced after the initial reaction of a ketose, e.g., d-fructose, yields 2-deoxy-2-aldosyllysine in two epimeric forms, i.e., glucosyl- and mannosyllysine (fructated lysine), which are both termed Heyns products. Both Amadori and Heyns products are prone to further reactions producing a class of heterogeneous compounds called advanced glycation endproducts (AGEs), which have been linked to the pathogenesis of diabetic complications, such as retinopathy, nephropathy, neuropathy, and accelerated atherosclerosis (Goh and Cooper 2008; Schalkwijk and Miyata 2012). Thus, Amadori and Heyns products appear to be promising precursors to monitor the formation of AGEs early. While a single glycation site can be quantified by ELISA, if a specific antibody is available, the large number of known glycation sites in serum or plasma proteins demands quantitative proteomics techniques, which typically rely on appropriate isotope-labeled standards. While synthetic Amadori peptides have been extensively used to study AGE formation (Greifenhagen et al. 2015) or to quantify prospective markers of type 2 diabetes (Spiller et al. 2017), only a few reports deal with d-fructose-modified (fructated) peptides. This was recently attributed to the challenging analysis of Heyns products and the Maillard reaction in general (Troise 2018). The lack of appropriate synthetic and analytical techniques is surprising, as fructation is more extensively involved in glycoxidation than glucation and, thus, represents an important research object (Hinton and Ames 2006; Semchyshyn 2013). As a consequence of increased sucrose or high-fructose corn syrup (HFCS) intake, preferentially consumed in the form of sugar-sweetened beverages, the fructose consumption raised markedly over the last 50 years (Bray et al. 2004). This excessive intake is associated with fatty liver (Jensen et al. 2018), metabolic syndrome, and type 2 diabetes (Malik and Hu 2015; Rodríguez et al. 2016). Fructose glycation may also contribute as a source of dietary AGEs, which can form in situ in the gastrointestinal tract (GI) lumen (DeChristopher 2017).

Initially, the site-specific modification of lysine residues by sugars used a global postsynthetic approach on solid phase (Frolov et al. 2006b). Thermal glucation of partially protected peptides provides good conversion rates, but requires long reaction times and high temperatures (70 °C or 110 °C) and purification is often challenging (Frolov and Hoffmann 2008). This approach was also applied to synthesize d-ribose and d-fructose-modified peptides to study their fragmentation behavior (Frolov et al. 2006a). However, poor yields were reported for fructation, which was also true for other sequences (Jakas et al. 2008). Similarly, the protocol reported by Heyns et al. (Heyns and Noack 1962) provided also low yields of fructated hippuryl-lysine using anhydrous conditions in DMSO at 75 °C (Krause et al. 2008). All three approaches yield a mixture of two epimers, i.e., 2-deoxy-2-glucosyl- and 2-deoxy-2-mannosyllysine, already reported in 1962 (Heyns and Noack 1962).

The weaknesses of postsynthetic glucation were overcome using protected sugars, i.e., 2,3:4,5-Di-O-isopropylidene-aldehydo-β-d-arabino-hexos-2-ulo-2,6-pyranose (Frolov et al. 2007), increasing the yields from ~ 30% to ~ 80%. Shortly afterwards, the synthesis of two building blocks, i.e., Fmoc-Lys(Fru,Boc)-OH and Fmoc-Lys(*i,i*-Fru,Boc)-OH, was reported (Carganico et al. 2009), which finally provides high yields for the site-specific synthesis of glucose-derived Amadori peptides (Stefanowicz et al. 2010). However, a similar straightforward building block is missing for routine synthesis of fructated peptides on solid phase.

Here, we describe a new solid-phase approach using a N^α^-Fmoc- and N^ε^-Boc-protected and N^ε^-fructated lysine for the site-specific synthesis of Heyns peptides containing epimerically pure 2-deoxy-2-glucosyllysine. The N^ε^-Boc-protection in combination with acidic purification conditions enabled a stereoselective conversion of the glucosyl-epimer. Finally, a fructated 15mer peptide corresponding to HSA Lys233 was synthesized on solid phase in high yield and purity. The stereoisomer configuration of glucosyllysine was confirmed by NMR spectroscopy.

## Experimental

### Reagents

Reagents were obtained from the following companies: AAPPTec LLC (Louisville, USA): Fmoc-l-Asp(O*t*Bu)-OH, Fmoc-l-Thr(*t*Bu)-OH and Fmoc-l-Val-OH; Biosolve B.V. (Valkenswaard, Netherlands): *N,N*-Dimethylformamide (DMF, > 99.8%) and piperidine (> 99.5%); Bruker Daltonik GmbH (Bremen, Germany): α-cyano-4-hydroxycinnamic acid (CHCA); Carl Roth GmbH (Karlsruhe, Germany): Acetic acid (ROTIPURAN 100%), acetone (ROTIPURAN ≥ 99.8%), dichloromethane (DCM, 99%), d-fructose (> 99.5%), pyridine (≥ 99.0%), trifluoroacetic acid (TFA, ≥ 99.9%, peptide synthesis grade) and zinc powder (≥ 98.0%); Fluka Analytical (Seelze, Germany): Acetic anhydride (≥ 99%), 1,3-diisopropyl-carbodiimide (DIC, ≥ 98.0%), Di-*tert*-butyldicarbonate (Boc_2_O), 1,2-ethandithiole (≥ 98%), *N,N*-Diisopropylethylamin (DIPEA) and thioanisole (≥ 99%); Iris Biotech GmbH (Marktredwitz, Germany): Fmoc-l-Leu-OH, Fmoc-l-Lys(Boc)-OH and Fmoc-l-Lys(Boc)-Wang-resin (100–200 mesh, loading capacity: 0.6 mmol/g); Merck KGaA (Darmstadt, Germany): Molecular sieve 4 Å; Orpegen Pharma GmbH (Heidelberg, Germany): Fmoc-l-Ala-OH monohydrate, Fmoc-l-Glu(O*t*Bu)-OH monohydrate, Fmoc-l-Phe-OH and Fmoc-l-Ser(*t*Bu)-OH; Sigma-Aldrich (Steinheim, Germany): Fmoc-l-Lysin hydrochloride (Fmoc-Lys-OH*HCl, ≥ 98.0%), *m*-cresol (98%), formic acid (FA, ~ 98%, LC–MS grade),1-hydroxy-benzotriazole hydrate (HOBt, ≥ 97.0%) and TFA (≥ 99%, HPLC grade); VWR International GmbH (Darmstadt, Germany): Acetonitrile (≥ 99.9%) and diethylether (99.9%). Water was purified in house (resistance ≥ 18 mΩ, total organic content < 1 ppb) with a PureLab Ultra Analytic System (ELGA Lab Water, Celle, Germany).

### Synthesis of N^α^-Fmoc-**l**-Lys[N^ε^-(2-deoxy-**d**-glucos-2-yl),N^ε^-Boc]-OH

**Fig. 1:**
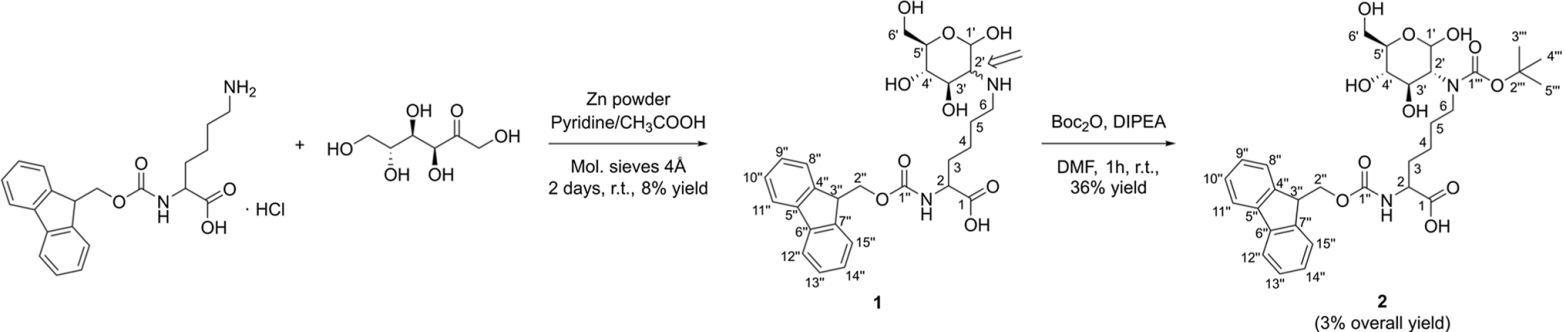
Reaction scheme of compound **2** (N^α^-Fmoc-Lys[N^ε^-(2-deoxy-d-glucos-2-yl),N^ε^-Boc]-OH. The arrow in structure **1** indicates an undefined stereocenter at C_2′_-position (wavy bond) corresponding to N^α^-Fmoc-Lys[N^ε^-(2-deoxy-d-glucos-2-yl)]-OH **1a** (equatorial position) and N^α^-Fmoc-Lys[N^ε^-(2-deoxy-d-mannos-2-yl)]-OH **1b** (axial position)

Fructation of N^α^-Fmoc-l-Lys was achieved according to the previously reported protocol (Srinivas and Harohally 2012) with slight modifications (Fig. 1). All reaction steps were performed at room temperature and under nitrogen atmosphere. Briefly, Fmoc-l-Lys·HCl (1.52 g, 3.77 mmol) and zinc powder (0.26 g, 4.00 mmol, 1.1 eq) were dissolved in a mixture of pyridine/glacial acetic acid (1:1 v/v, 20 mL, dried over 4 Å molecular sieve) and stirred for 40 min. d-fructose (0.81 g, 4.47 mmol, 1.2 eq) and fresh molecular sieve (4 Å, 1.50 g) were added and the suspension gently stirred for 2 days. The suspension was filtered and the solid product washed thoroughly twice with pyridine/glacial acetic acid (1:1 v/v, 2.5 mL). The combined filtrate was split in six aliquots of around 4 mL in polypropylene tubes and ice-cold acetone (46 mL) added to each tube. Samples were incubated (− 20 °C, 2 days) and centrifuged (3000×g, 5 min, 4 °C, Allegra centrifuge 21R; Beckman Coulter, Krefeld, Germany). The supernatants were discarded and each pellet, washed with ice-cold acetone (15 mL), and dried in vacuum (0.1 mbar) to obtain a beige product (0.60 g, 1.13 mmol, 30% yield).

An aliquot of the crude product (133 mg) was reconstituted in 30% (v/v) aqueous acetonitrile containing 0.1% (v/v) formic acid and centrifuged (3030×g, 5 min, RT, Centrifuge 5702; Eppendorf AG, Hamburg, Germany). The supernatant was purified by reversed-phase high-performance liquid chromatography (RP-HPLC), as described below, and fractions corresponding to the expected molecular mass were lyophilized. The purified product (36.9 mg, 8% yield) was obtained as a mixture of epimers Fmoc-l-Lys(Glc)-OH **1a** and Fmoc-l-Lys(Man)-OH **1b** as white solid, which will be referred to as Fmoc-l-Lys(Glc/Man)-OH **1**. A second purification of the crude product (125 mg) by RP-HPLC collecting only the early eluting fraction yielded the pure glucosyl-epimer Fmoc-Lys(Glc)-OH **1a** as a brown, crystalline solid after lyophilization (12.0 mg, 98% purity according to UV-HPLC, 3% yield).

N^α^-Fmoc-Lys[N^ε^-(2-deoxy-d-glucos/mannos-2-yl)]-OH (**1**).

***HRMS: ***ESI-MS (positive ion mode): [C_27_H_34_N_2_O_9_ + H]^+^ at *m/z* 531.2335 (Theory: *m/z* 531.2337).

MP = 113–114 °C (caramelization and decomposition observed). ${[{\alpha }]}_{\text{D}}^{22.5}$ =  + 15.97 (*c* = 0.1, H_2_O).

***NMR***: ^1^H-NMR (700 MHz, DMSO-*d*_6_) δ (ppm) = 7.90 (d, *J* = 7.6 Hz, 2H, 11″, 12″-C_Ar_**H**), 7.72 (t, *J* = 7.1 Hz, 2H, 8″,15″-C_Ar_**H**), 7.67–7.60 (m, 1H, N**H**), 7.43 (t, *J* = 7.5 Hz, 2H, 9″,14″-C_Ar_**H**), 7.34 (t, *J* = 7.5 Hz, 2H, 10″,13″-C_Ar_**H**), 5.37 (d, *J* = 5.0 Hz, 1′-C**H, α-mannofuranosyl**), 5.32 (d, *J* = 3.6 Hz, 1′-C**H**_**eq**_**, α-glucopyranosyl**), 5.28 (d, *J* = 2.1 Hz, 1′-C**H**_**eq**_**, α-mannopyranosyl**), 4.99 (d, *J* = 1.4 Hz, 1′-C**H**_**ax**_, **β-mannopyranosyl**), 4.77 (d, *J* = 8.3 Hz, 1′-C**H**_**ax**_, **β-glucopyranosyl**), 4.37–4.26 (m, 2H, 2″-C**H**_**2**_), 4.23 (t, *J* = 7.1 Hz, 1H, 3″-C**H**), 3.93 (dd, *J* = 9.7, 4.7 Hz, 1H, 2-C**H**), 3.74–3.69 (m, 1H, 3′-C**H**), 3.64 (dd, *J* = 12.1, 2.2 Hz, 1H, 6′-C**H**H), 3.61–3.57 (m, 1H, 5′-C**H**), 3.25–3.15 (m, 1H, 4′-C**H**), 3.02 (dt, *J* = 11.4, 5.8 Hz, 1H, 6-C**H**H), 2.97 (dd, *J* = 10.4, 3.7 Hz, 1H, 2′-C**H**), 2.93 (dt, *J* = 11.7, 5.8 Hz, 1H, 6-CH**H**), 1.76–1.52 (m, 4H, 3-C**H**_**2**_, 5-C**H**_**2**_), 1.39–1.26 (m, 2H, 4-C**H**_**2**_).

N^α^-Fmoc-Lys[N^ε^-(2-deoxy-d-glucos-2-yl)]-OH (**1a**).

***MS:*** MALDI- and ESI-MS (positive ion mode): [C_27_H_34_N_2_O_9_ + H]^+^ at *m/z* 531.13 and at *m/z* 531.09, respectively (Theory: *m/z* 531.23).

$${[{\alpha }]}_{\text{D}}^{22.5}$$ =  + 20.00 (*c* = 0.1, H_2_O).

***NMR***: ^1^H-NMR (700 MHz, DMSO-*d*_6_) δ (ppm) = 7.89 (d, *J* = 7.4 Hz, 2H, 11″, 12″-C_Ar_**H**), 7.71 (t, *J* = 7.2 Hz, 2H, 8″,15″-C_Ar_**H**), 7.63 (m, 1H, N**H**), 7.43 (t, *J* = 7.4 Hz, 2H, 9″,14″-C_Ar_**H**), 7.34 (t, *J* = 7.4 Hz, 2H, 10″,13″-C_Ar_**H**), 5.31 (d, *J* = 3.5 Hz, 1′-C**H**_**eq**_**, α-pyranosyl 83%**), 4.76 (d, *J* = 8.3 Hz, 1′-C**H**_**ax**_, **β-pyranosyl 17%**), 4.37–4.26 (m, 2H, 2″-C**H**_**2**_), 4.22 (t, *J* = 7.0 Hz, 1H, 3″-C**H**), 3.93 (dd, *J* = 9.7, 4.7 Hz, 1H, 2-C**H**), 3.74–3.69 (m, 1H, 3′-C**H**), 3.64 (dd, *J* = 12.1, 2.2 Hz, 1H, 6′-C**H**H), 3.61–3.57 (m, 1H, 5′-C**H**), 3.20–3.15 (m, 1H, 4′-C**H**), 3.02 (dt, *J* = 11.4, 5.8 Hz, 1H, 6-C**H**H), 2.97 (dd, *J* = 10.4, 3.7 Hz, 1H, 2′-C**H**), 2.93 (dt, *J* = 11.7, 5.8 Hz, 1H, 6-CH**H**), 1.76–1.52 (m, 4H, 3-C**H**_**2**_, 5-C**H**_**2**_), 1.39–1.26 (m, 2H, 4-C**H**_**2**_).

### N^α^-Fmoc-**L**-Lys[N^ε^-(2-deoxy-**d**-glucos-2-yl),N^ε^-Boc]-OH (Fmoc-Lys(Glc,Boc)-OH; 2).

Pure Fmoc-Lys(Glc/Man)-OH **1** (43.2 mg, 81.4 µmol) was dissolved in DMF (2.9 mL) before Boc_2_O (374 µL, 1.63 mmol, 20 eq.) and DIPEA (69.7 µL, 0.41 mmol, 5 eq.) were added. The reaction mixture was stirred at room temperature. After 1 h, the reaction was stopped by consecutively adding 60% (v/v) aqueous acetonitrile containing 0.1% TFA (8.35 mL) and 0.1% (v/v) aqueous TFA (8.35 mL). The solution was purified by RP-HPLC and lyophilized yielding **2** (18.5 mg, 29.3 µmol, 96% purity according to UV-HPLC, 36% yield) as a white solid. As a byproduct N^α^-Fmoc-Lys[N^ε^-(2-deoxy-d-mannos-2-yl)]-OH **1b** (0.58 mg, 1.09 µmol, 92% purity according to UV-HPLC) was obtained as a white solid, contaminated by N^α^-Fmoc-Lys[N^ε^-(2-deoxy-d-glucos-2-yl)]-OH (8% according to UV-HPLC).

N^α^-Fmoc-Lys[N^ε^-(2-deoxy-d-glucos-2-yl),N^ε^-Boc]-OH (**2**).

***HRMS: ***ESI-MS (negative ion mode): [C_32_H_42_N_2_O_11_–H]^–^ at *m/z* 629.2721 (Theory: *m/z* 629.2716).

MP = 120–124 °C (decomposition observed). ${[{\alpha }]}_{\text{D}}^{22.5}$ =  + 16.1 (*c* = 0.1, MeOH).

***MS:*** ESI-MS (negative ion mode): [C_32_H_42_N_2_O_11_–H]^–^ at *m/z* 628.94 (Theory: *m/z* 629.27).

***NMR***: ^1^H-NMR (700 MHz, DMSO-*d*_6_) δ (ppm) = 7.89 (d, *J* = 7.5 Hz, 2H, 11″, 12″-C_Ar_**H**), 7.72 (t, *J* = 6.5 Hz, 2H, 8″,15″-C_Ar_**H**), 7.63 (m, 1H, N**H**), 7.42 (t, *J* = 7.4 Hz, 2H, 9″,14″-C_Ar_**H**), 7.34 (t, *J* = 7.5 Hz, 2H, 10″,13″-C_Ar_**H**), 6.03 (d, *J* = 5.5 Hz), 5.83 (d, *J* = 6.7 Hz, 1′-C**H**_**eq**_, Fmoc-Lys(Man,Boc)), 5.73 (d, *J* = 6.1 Hz), 5.31 (d, *J* = 3.5 Hz), 5.27 (d, *J* = 2.2 Hz), 5.15 (s), 4.99 (d, *J* = 7.8 Hz, 1′-C**H**_**ax**_, **β-pyranosyl** Fmoc-Lys(Glc,Boc)), 4.92 (d, *J* = 3.0 Hz), 4.87 (d, *J* = 3.3 Hz, 1′-C**H**_**eq**_, **α-pyranosyl** Fmoc-Lys(Glc,Boc)), 4.75 (d, *J* = 8.2 Hz), 4.67–4.63 (m), 4.56 (d, *J* = 8.5 Hz), 4.34–4.25 (m, 2H, 2″-C**H**_**2**_), 4.23 (t, *J* = 7.0 Hz, 1H, 3″-C**H**), 4.18 (d, *J* = 2.6 Hz), 4.09 (d, *J* = 5.6 Hz), 3.93 (td, *J* = 8.7, 8.2, 4.5 Hz, 1H, 2-C**H**), 3.89 (dd, *J* = 9.7, 4.8 Hz), 3.84 (dd, *J* = 10.4, 8.0 Hz, 1H, 2′-C**H**), 3.76–3.70 (m, 1H, 3′-C**H**), 3.70–3.58 (m, 1H, 6′-C**H**H), 3.56 (dd, *J* = 11.4, 2.6 Hz, 1H, 5′-C**H**), 3.24–3.07 (m, 2H, 6-C**H**_**2**_), 3.05–2.99 (m, 1H, 4′-C**H**), 2.50–2.47 (m, 1H, 2′-C**H**), 1.76–1.56 (m, 4H, 3-C**H**_**2**_, 5-C**H**_**2**_), 1.55–1.34 (m, 9H,C(C**H**_**3**_)_3_) 1.33–1.17 (m, 2H, 4-C**H**_**2**_).

The presence of residual Fmoc-Lys(Glc)-OH and Fmoc-Lys(Man)-OH leads to additional signals at 5.31 (d, *J* = 3.5 Hz, 1′-C**H**_**eq**_**, α-pyranosyl**) and 4.75 (d, *J* = 8.2 Hz, 1′-C**H**_**ax**_, **β-pyranosyl**), as well as 5.27 (d, *J* = 2.2 Hz, 1′-C**H**_**eq**_, **α-pyranosyl**) and 4.92 (d, *J* = 3.0 Hz, 1′-C**H**_**ax**_, **β-pyranosyl**).

(N^α^-Fmoc-Lys[N^ε^-(2-deoxy-d-mannos-2-yl)]-OH (**1b**).

***MS: ***ESI-MS (negative ion mode): [C_29_H_34_N_2_O_9_–H]^–^ at *m/z* 528.89 (Theory: *m/z* 529.23).

***NMR: ***^1^H-NMR (700 MHz, DMSO-*d*_6_) δ (ppm) = 7.90 (d, *J* = 7.5 Hz, 2H, 11″, 12″-C_Ar_**H**), 7.72 (t, *J* = 7.0 Hz, 2H, 8″,15″-C_Ar_**H**), 7.62 (m, 1H, N**H**), 7.43 (t, *J* = 7.4 Hz, 2H, 9″,14″-C_Ar_**H**), 7.34 (t, *J* = 7.4 Hz, 2H, 10″,13″-C_Ar_**H**), 5.30 (d, *J* = 3.7 Hz, 1′-C**H, α-glucopyranosyl + α-mannofuranosyl 21%**), 5.29 (d, *J* = 5.5 Hz, 1′-C**H**, **β-mannofuranosyl 8%**), 5.21 (s, 1′-C**H**_**eq**_, **α-mannopyranosyl 39%**), 4.96 (s, 1′-C**H**_**ax**_, **β—mannopyranosyl 32%**), 4.35–4.26 (m, 2H, 2″-C**H**_**2**_), 4.23 (t, *J* = 7.1 Hz, 1H, 3″-C**H**), 3.93 (dd, *J* = 9.7, 4.7 Hz, 1H, 2-C**H**), 3.88–3.85 (m, 1H, 3′-C**H**), 3.67–3.64 (m, 1H, 6′-C**H**H), 3.64–3.60 (m, 1H, 5′-C**H**), 3.54 (dd, *J* = 11.5, 5.9 Hz, 1H, 6′-CH**H**), 3.35 (dd, *J* = 10.3, 7.5 Hz, 1H, 4′-C**H**), 3.14–3.08 (m, 1H, 2′-C**H**), 3.04 (dd, *J* = 10.2, 6.3 Hz, 1H, 6-C**H**H), 2.98–2.82 (m, 1H, 6-CH**H**), 1.76–1.52 (m, 4H, 3-C**H**_**2**_, 5-C**H**_**2**_), 1.38–1.29 (m, 2H, 4-C**H**_**2**_).

The presence of residual Fmoc-Lys(Glc)-OH leads to additional signals at 5.30 (d, *J* = 3.7 Hz).

### Solid-phase peptide synthesis

Peptides AEFAEVSKLVTDLTK either containing N^ε^-glucosyllysin (**3a**) or unmodified lysine (**3b**) at position 8 were synthesized using the 9-fluorenylmethoxy-carbonyl/*tert*-butyl (Fmoc/^*t*^Bu) strategy (25-µmol scale), DIC/HOBT activation (8 eq.) in DMF, and Fmoc-l-Lys(Boc)-Wang resin (100–200 mesh, loading capacity: 0.6 mmol/g) on a multiple synthesizer (SYRO2000, MultiSynTech GmbH, Witten, Germany). The Fmoc-group was cleaved with piperidine in DMF (40% v/v for 3 min and 20% v/v for 10 min). Fmoc-Lys(Glc,Boc)-OH (**2**, 10.5 mg, 16.7 µmol, 2 eq.) was coupled manually to the peptidyl resin (8.3 µmol, 1.5 h) followed by endcapping with acetic anhydride in DMF (20%, v/v, 20 min) before the synthesis was continued on the peptide synthesizer. Peptides were cleaved with TFA containing a scavenger mixture (12.5% v/v; 1,2-ethandithiole, m-cresol, thioanisole, and water; 1/2/2/2, v/v/v/v) for 3 h, precipitated with cold diethyl ether, washed twice with diethyl ether, and dried.

### Purification and characterization

Lysine derivatives and peptides were purified on an Äkta Purifier 10 (GE Healthcare Europe GmbH, Freiburg, Germany) using Jupiter C_18_-columns (Phenomenex, Inc., Torrance, U.S.A.) with inner diameters (ID) of either 21.2 mm (length: 250 mm, particle size: 15 µm, pore size: 300 nm) or 10 mm (length: 250 mm, particle size: 5 µm, pore size: 300 nm). Eluent A was 0.1% (v/v) aqueous TFA and eluent B was 60% (v/v) aqueous acetonitrile containing 0.1% (v/v) TFA. Purifications relied on linear gradients with a slope of 0.6% acetonitrile per minute.

Melting points were determined in capillary tubes using a Büchi M-560 (BÜCHI Labortechnik GmbH, Essen, Germany) without corrections. Optical rotations were measured on a Polarotronic polarimeter (Schmidt & Haensch GmbH & Co., Berlin, Germany). Monoisotopic masses were determined by matrix-assisted laser desorption/ionization time-of-flight mass spectrometry (MALDI-TOF/TOF-MS) on a 5800 proteomic analyzer (ABSciex GmbH, Darmstadt, Germany) operating in reflector mode and using a solution of α-cyano-4-hydroxycinnamic acid (4 g/L) in eluent B as matrix.

Samples were analyzed by RP-HPLC on an 1100 LC system (Agilent Technologies, Santa Clara, CA, USA) equipped with an UV detector (absorbance recorded at 214 nm and 301 nm) coupled online to an ion trap mass spectrometer equipped with an electrospray ionization source (ESI-IT-MS, Esquire HCT, Bruker Daltonics) operated in positive or negative ion mode. Separations relied on a Jupiter C_18_-column (ID: 2 mm, length: 150 mm, particle size: 5 µm, pore size: 300 Å, Phenomenex) using a column temperature of 60 °C and a flow rate of 0.2 mL/min. Eluents were 0.1% (v/v) aqueous formic acid (eluent C) and 60% (v/v) aqueous acetonitrile containing 0.1% (v/v) formic acid (eluent D). Gradients used a linear slope from 5 to 95% eluent D in 30 min. The ESI source was operated at a source temperature of 365 °C using nitrogen as curtain gas (40 psi) and dry gas (9 L/min).

Peptides modified by glucose or fructose were analyzed on a nanoAcquity UPLC (Waters GmbH) coupled online to a Synapt G2-S*i* mass spectrometer (Waters GmbH) equipped with a nanoESI source (Waters GmbH) operated in positive ion mode. Eluents were eluent C and acetonitrile containing 0.1% (v/v) formic acid (eluent E). Peptides were trapped on a nanoAcquity Symmetry C18-column (ID: 180 μm, length: 2 cm, particle diameter: 5 μm) at a flow rate of 5 μL/min (3% eluent E). Separation was achieved on a BEH 130 column (C18-phase, ID: 75 μm, length: 10 cm, particle diameter: 1.7 μm) using a flow rate of 0.35 μL/min and a column temperature of 35 °C. Peptides were eluted with linear gradients from 3% E to 40% eluent E in 19 min and to 85% eluent E in 5 min. Tandem mass spectra were recorded in data independent acquisition (DIA, MS^E^) mode using the following settings: capillary voltage 3 kV, sampling cone of 30 kV, source offset of 60 kV, source temperature of 80 °C, cone gas flow of 20 L/h, nano-flow gas pressure of 0.20 bar, and a scan time of 0.5 s. Ions were fragmented in the trap cell using a collision energy ramp from 18 to 50 V.

### NMR

All NMR spectra were recorded on a 700 MHz Bruker Avance III spectrometer equipped with a cryocooled TCI probe at 298 K. The ^1^H-NMR spectra were recorded over a spectral width of 25 ppm. The free induction decay (FID) was digitized with 64 k data points and multiplied by an exponential window function (lb = 0.3) prior to Fourier transformation. All ^1^H chemical shifts are referenced to DMSO-*d*_5_ (δ = 2.50 ppm). The HSQC spectra (pulse program: *hsqcedetgpsisp2.4*) were recorded with a (F2, F1) resolution of (1024, 256) data points over a spectral width of (12 ppm, 165 ppm). The data points were zero-filled to (1024, 1024) points and multiplied by a qsine (SSB 2, SSB 2) window function prior to Fourier transformation. The HMBC spectra (pulse program: *hmbcgpl2ndqf*) were recorded with a (F2, F1) resolution of (1024, 512) data points over a spectral width of (12 ppm, 230 ppm). The data points were zero-filled to (1024, 1024) points and multiplied by a qsine (SSB 2, SSB 0) window function prior to Fourier transformation. The COSY spectra (pulse program: *cosygpqf*) were recorded with a (F2, F1) resolution of (1024, 512) data points over a spectral width of (12 ppm, 12 ppm). The data points were zero-filled to (1024, 1024) points and multiplied by a qsine (SSB 0, SSB 0) window function prior to Fourier transformation.

## Results and discussion

### Synthesis of the d-fructose-modified lysine building block Fmoc-Lys(Glc,Boc)-OH

**Fig. 2:**
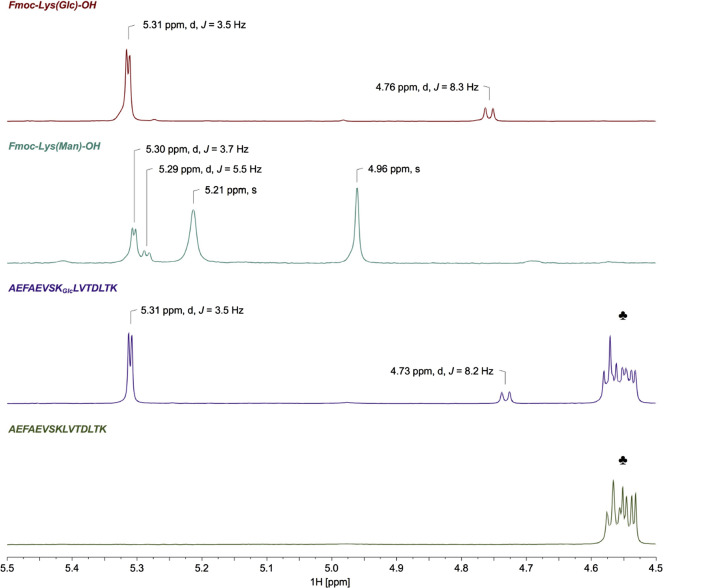
Zoomed proton NMR spectra (4.5–5.5 ppm) of purified N^α^-Fmoc-Lys[N^ε^-(2-deoxy-d-glucos-2-yl)]-OH (**1a**), N^α^-Fmoc-Lys[N^ε^-(2-deoxy-d-mannos-2-yl)]-OH (**1b**), AEFAEVSK_Glc_LVTDLTK (**3a**) and AEFAEVSKLVTDLTK (**3b**) from top to bottom. Chemical shifts and coupling constants of the proton at C_1_′ are labeled, allowing conclusions about the sugar modification and its anomeric equilibrium. Peptide-derived signals (4.60–4.51 ppm, m) are marked with spades (♣)

**Fig. 3:**
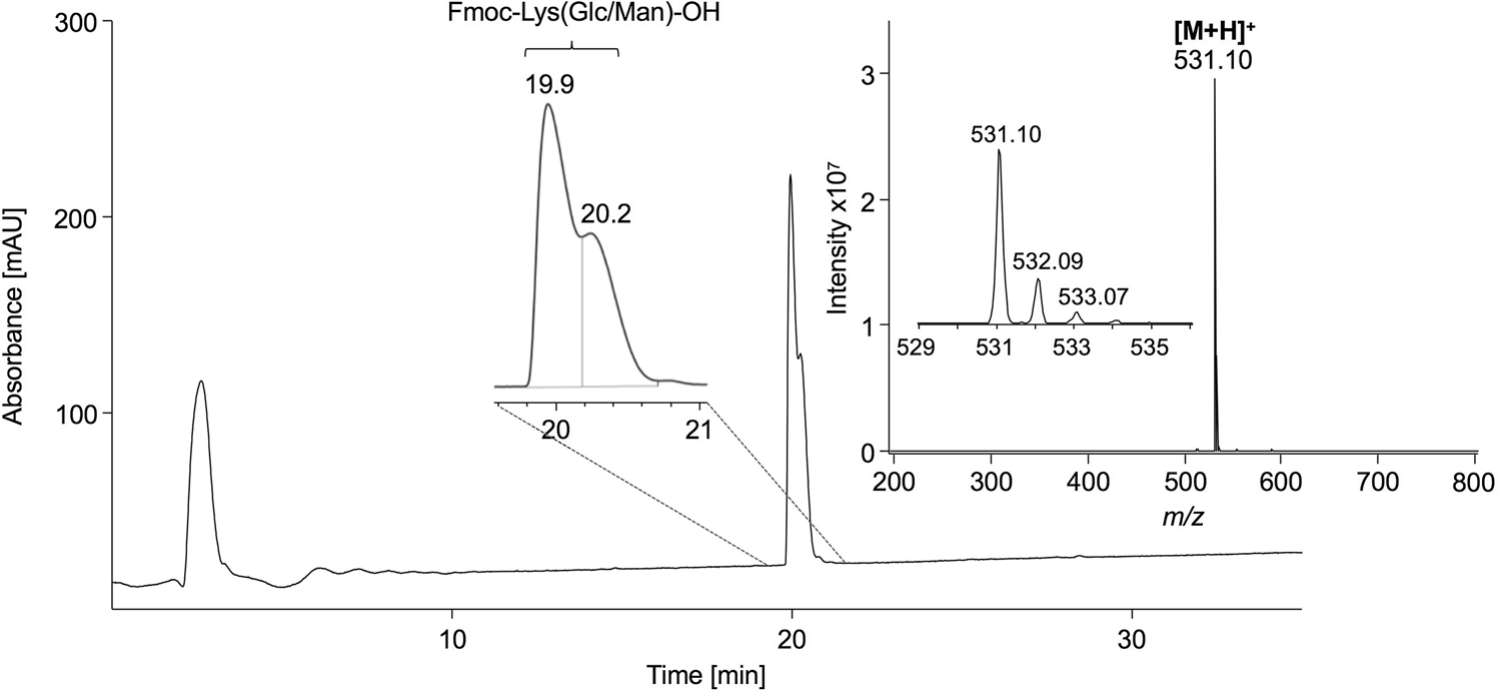
Chromatogram and the ESI-MS (insert) of purified N^α^-Fmoc-Lys[N^ε^-(2-deoxy-d-glucos/mannos-2-yl)]-OH (**1**). The compound was analyzed by RP-HPLC using a linear 30-min gradient from 3 to 57% aqueous acetonitrile containing 0.1% formic acid (absorbance recorded at 214 nm). The mass spectrum from 19.4 to 21.9 min was recorded online on an ESI-ion trap-MS in positive ion mode from *m/z* 200 to 800. The small insert shows the isotope pattern of the protonated quasimolecular ion. Further details are provided in methods and the corresponding NMR spectrum is shown in Supplement, Fig. S6

The synthesis of glycated peptides in high yields and purities requires building blocks suitable for peptide synthesis. While glucated peptides can be routinely synthesized by the Fmoc/^*t*^Bu-strategy using protected building blocks (Carganico et al. 2009; Stefanowicz et al. 2010), a suitable strategy for the synthesis of fructated peptides containing N^ε^-glucosyl- or N^ε^-mannosyllysine is still missing. Therefore, we synthesized a building block based on a N^ε^-fructose-modified Fmoc-Lys(Boc)-OH (Fig. 1). Fmoc-l-Lys-OH was glycated adopting a method reported for fructation of lysine (Srinivas and Harohally 2012), which yields a mixture of l-Lys(Glc) and l-Lys(Man). Fmoc-Lys-OH, which was well soluble and stable in the aprotic solvent system, remained in the crude product (35%) besides two partially separated substances eluting on RP-HPLC at 20.3 min (39%) and 20.7 min (19%). Both products displayed the same monoisotopic mass of 531.1 Da (calculated 531.2 Da) indicating glucosyl- and mannosyl-epimers (Supplement, Fig. S1). Presumably, the earlier eluting species represented the glucosyl-epimer, as its formation is thermodynamically favored due to the equatorial position of the bulky lysine residue (Heyns et al. 1967). Tandem mass spectra acquired on a MALDI-TOF/TOF-MS displayed the typical fragmentation pattern reported for d-fructose-modified peptides (Supplement, Fig. S2) (Frolov et al. 2006a). Preparative RP-HPLC yielded glucosyllysine (**1a**) with a high purity of 98% (Supplement, Fig. S3), whereas the fraction potentially containing mannosyllysine (**1b**) was contaminated by similar quantities of glucosyllysine (Supplement, Fig. S4). The identity of Fmoc-Lys(Glc)-OH (**1a**) was confirmed by ^1^H-NMR (Supplement, Fig. S5), with the spectrum showing sugar-derived signals from 4.5 ppm to 5.5 ppm (Fig. 2, first panel). The well-defined doublets at 4.76 ppm (*J* = 8.3 Hz) and 5.31 ppm (*J* = 3.5 Hz) are characteristic for β- and α-glucopyranosyl forms, respectively. All signals are in good agreement with previous reports (Jakas et al. 2008; Krause et al. 2008; Srinivas and Harohally 2012; Treibmann et al. 2017; Ohno et al. 2019). Based on the peak areas of the anomeric proton signals, the glucosyl derivative was present in α-pyranose (83%) and β-pyranose (17%) forms. The observed shifts and couplings of anomeric protons together with the chromatographic data indicated the successful isolation of the glucosyl-epimer. A second aliquot crude product was purified and fractions containing both glucosyl- and mannosyl-Fmoc-Lys (Fmoc-Lys(Glc/Man)-OH, **1**) were combined (Fig. 3, 8% yield). The ^1^H-NMR spectrum displayed in addition to the signals of the glucosyl-epimer, further C1′-proton signals resolved as doublets at 4.99 ppm (*J* = 1.4 Hz), 5.28 ppm (*J* = 2.1 Hz) and 5.37 ppm (*J* = 5.0 Hz) (Supplement, Fig. S6). The two upfield shifted signals indicate the β- and α-mannopyranosyl forms, whereas the latter one represents the α-mannofuranosyl form.

**Fig. 4:**
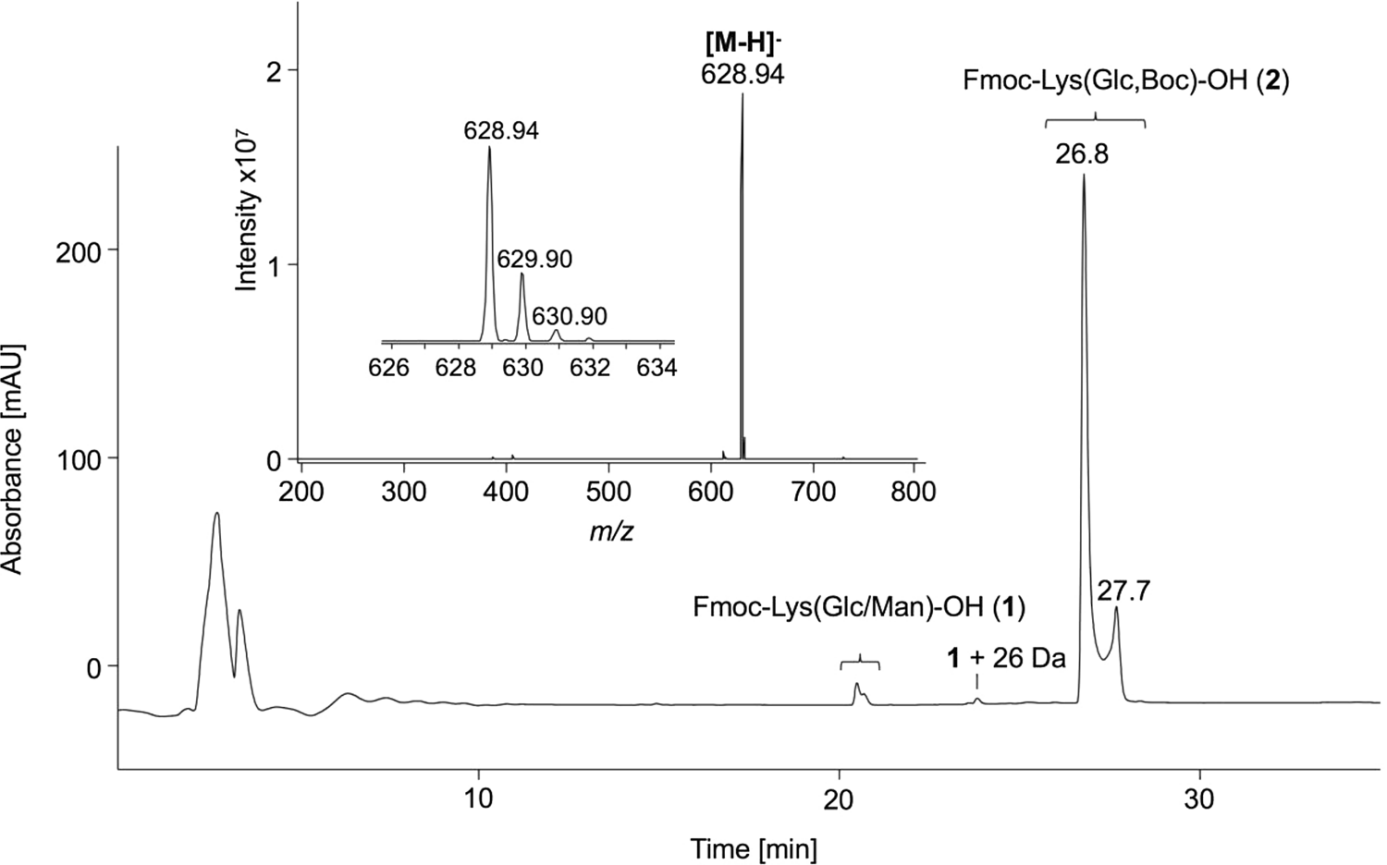
Chromatogram and the ESI-MS (insert) of purified N^α^-Fmoc-Lys[N^ε^-(2-deoxy-d-glucos-2-yl),N^ε^-Boc]-OH) (**2**). The compound was analyzed by RP-HPLC using a linear 30-min gradient from 3 to 57% aqueous acetonitrile containing 0.1% formic acid (absorbance recorded at 214 nm). The mass spectrum from 26.5 to 28.0 min was recorded online on an ESI-ion trap-MS in negative ion mode from *m/z* 200 to 800. The small insert shows the isotope pattern of the deprotonated quasimolecular ion. Acidic Boc-hydrolysis leads to trace amounts of earlier eluting glucosyl- (20.5 min) and mannosyllysine (20.7 min), as well as an unknown contaminant (23.8 min) with a mass increase of + 26 Da referred to the starting material. Further details are provided in methods and the corresponding NMR is shown in Supplement, Fig. S9

As the glycated ε-amino group was partially acylated during SPPS (data not shown), a Boc-protecting group was introduced using the purified mixture Fmoc-Lys(Glc/Man)-OH (**1**). The reaction monitoring by RP-HPLC in the presence of 0.1% (v/v) TFA as ion pair reagent indicated that the first peak corresponding to Fmoc-Lys(Glc)-OH disappeared after one hour, while a new signal appeared around 6 min later (21% of all peak areas, Supplement, Fig. S7). Surprisingly, the area of the second peak remained stable even after two hours. When the same reaction mixture was separated with formic acid as ion pair reagent by LC–MS, the peak area of Fmoc-Lys(Man)-OH peak was reduced after 2 h (Supplement, Fig. S8), while a second Boc-protected product peak appeared. Due to the protection of both amino groups, the mass spectra were recorded in negative ion mode. They confirmed isobaric products at three different retention times. After purification by RP-HPLC (TFA as ion pair reagent), the presumed Fmoc-Lys(Glc,Boc)-OH (**2**) was obtained with a yield of 36% and a purity of 96% according to UV-HPLC (eluents containing formic acid); the overall yield was 3%. The monoisotopic mass of 630.28 Da was confirmed by ESI-MS in negative ion mode (629.95 Da, Fig. 4). The peak areas indicated that the fraction at 26.8 min (76%) contained most likely the α-glucopyranose form and the fraction at 27.7 min probably the β-glucopyranose form (20%) considering the ratios of the tautomeric forms of Fmoc-Lys(Glc)-OH (**1a**) (Fig. 2, first panel). The recorded NMR showed multiple signal sets due to rotatory isomers from 1.34 to 1.55 ppm representing the aliphatic protons of a *tert*-butyl group (Supplement, Fig. S9). The anomeric proton region (4.5–6.1 ppm) was very crowded making it difficult to annotate the sugar signals. Most likely the lyophilized fraction contained trace amounts of earlier eluting glucosyl- (20.5 min, 2%) and mannosyllysine (20.7 min, 1%) as well as an unknown byproduct (23.8 min, 1%) detected with a 26 Da higher mass than Fmoc-Lys(Glc/Man)-OH. This may indicate the hydrolysis of the Boc-group, which appears highly pH-dependent, during RP-HPLC, as different conversion rates of the product were determined for different ion pair reagents (Supplement, Fig. S7/S8). Fractions eluting late from preparative column implied that preferentially Fmoc-Lys(Man,Boc)-OH was prone to hydrolysis in TFA-containing eluents (Supplement, Fig. S10). The reaction of the manno-epimer might be favored by electrostatic interactions between preferentially the 3-OH or 6-OH groups and the neighbored Boc-carbonyl group. This may enhance its electrophilic character promoting Boc-hydrolysis by a nucleophilic attack of water. The use of amino sugar-derived organocatalysts increasing the enantioselectivity of a reaction and to activate carbonyls due to hydrogen bonding with hydroxyl groups was reported for asymmetric organic syntheses (Singh et al. 2008; Agarwal and Peddinti 2011). Importantly, purification of Fmoc-Lys(Man,Boc)-OH appears possible, but low pH conditions may promote its decomposition. The NMR of Fmoc-Lys(Glc)-OH presented above supports a separation of tautomers, as only the α- and β-pyranose forms were present, explaining the signals observed in RP-HPLC. Similarly, Carganico et al. observed four peaks for the Amadori analog N^α^-Fmoc-Lys[N^ε^-1-deoxy-d-fructos-1-yl,N^ε^-Boc)]-OH, which were explained by four tautomeric forms resulting from mutarotation of the sugar moiety (Carganico 2010).

The purification of Fmoc-Lys(Glc,Boc)-OH provided the unreacted Fmoc-Lys(Man)-OH in high purity (92%, Supplement, Fig. S11). The NMR spectrum of Fmoc-Lys(Man)-OH (Supplement, Fig. S12) was very similar to Fmoc-Lys(Glc,Boc)-OH and Fmoc-Lys(Glc)-OH, but displayed characteristic differences from 4.5 to 5.5 ppm (Fig. 2, second panel). The singlets at 4.96 ppm and 5.21 ppm were assigned to β- and α-mannopyranosyl forms, respectively, and the doublet at 5.29 ppm (*J* = 5.5 Hz) to the β-mannofuranosyl tautomer. The upshifted signal at 5.30 ppm (*J* = 3.7 Hz) is supposed to correspond to the α-mannofuranosyl tautomer (Treibmann et al. 2017). The difference in the coupling constant was attributed to an overlay with the α-glucopyranosyl form of the Fmoc-Lys(Glc)-OH impurity (**1a**, 8%). All observed signals are in accordance with previous reports (Treibmann et al. 2017). Based on the peak areas of the anomeric proton signals, the α- and β-mannopyranosyl (39% and 32%) forms are dominant, whereas the α- and β-mannofuranosyl (21% and 8%) are present at a lower extent.

## Peptide synthesis

**Fig. 5:**
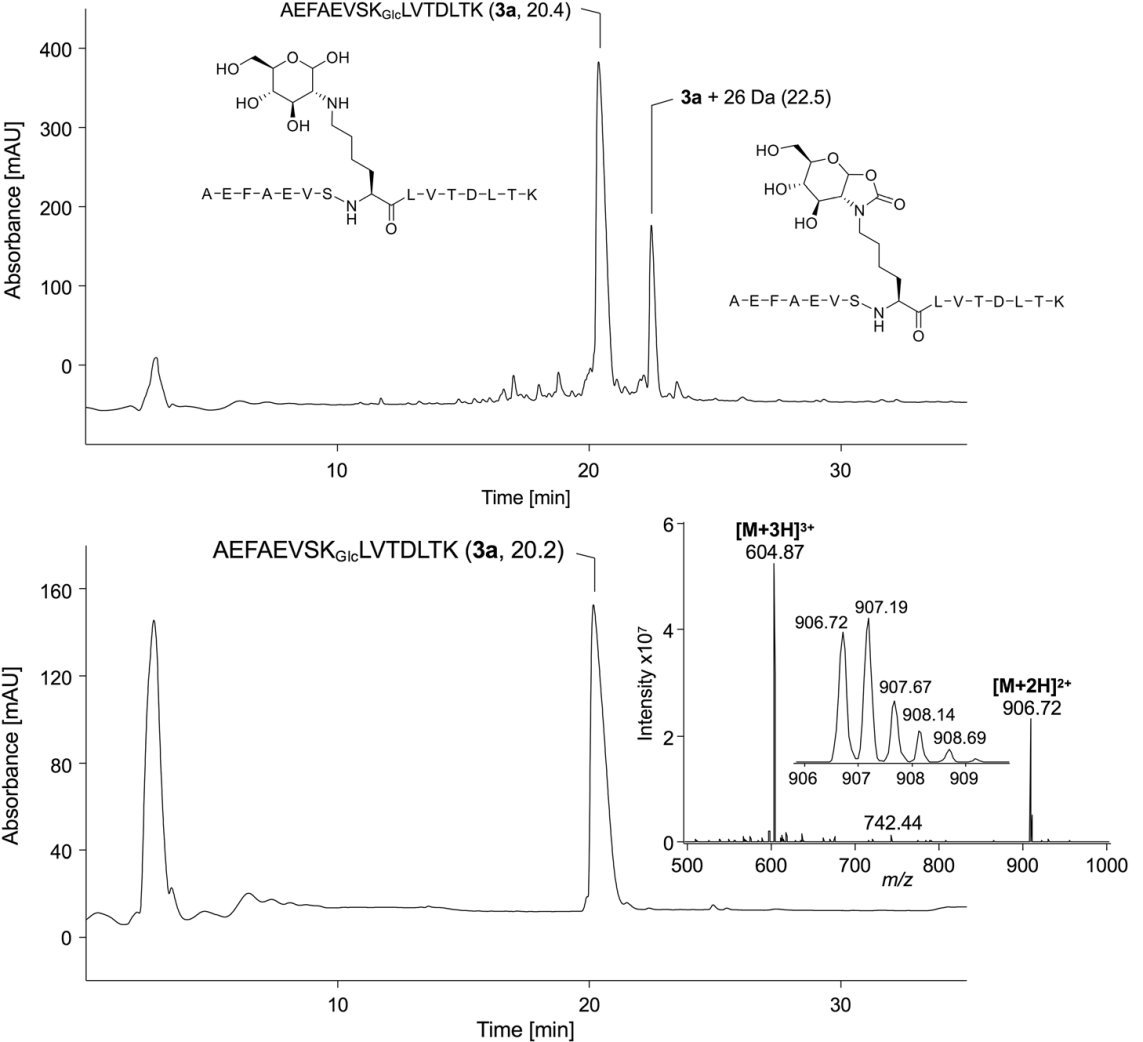
Chromatograms and the ESI-MS (insert) of crude (top) and purified (bottom) AEFAEVSK_Glc_LVTDLTK (**3a**). Compounds were analyzed by RP-HPLC using a linear 30-min gradient from 3 to 57% and a linear 20-min gradient from 24 to 36% aqueous acetonitrile containing 0.1% formic acid, respectively (absorbance recorded at 214 nm). The mass spectrum from 20.0 to 22.0 min was recorded online on an ESI-ion trap-MS in positive ion mode from *m/z* 500 to 1000. The small insert shows the isotope pattern of the doubly protonated quasimolecular ion at *m/z* 906.72. The observed byproduct (22.5 min) is described by its proposed structure, assuming an irreversible rearrangement in the context of Boc-group cleavage (see Fig. S14). Further details are provided in methods and the full mass spectra of crude **3a** are shown in Supplement, Fig. S13

**Fig. 6:**
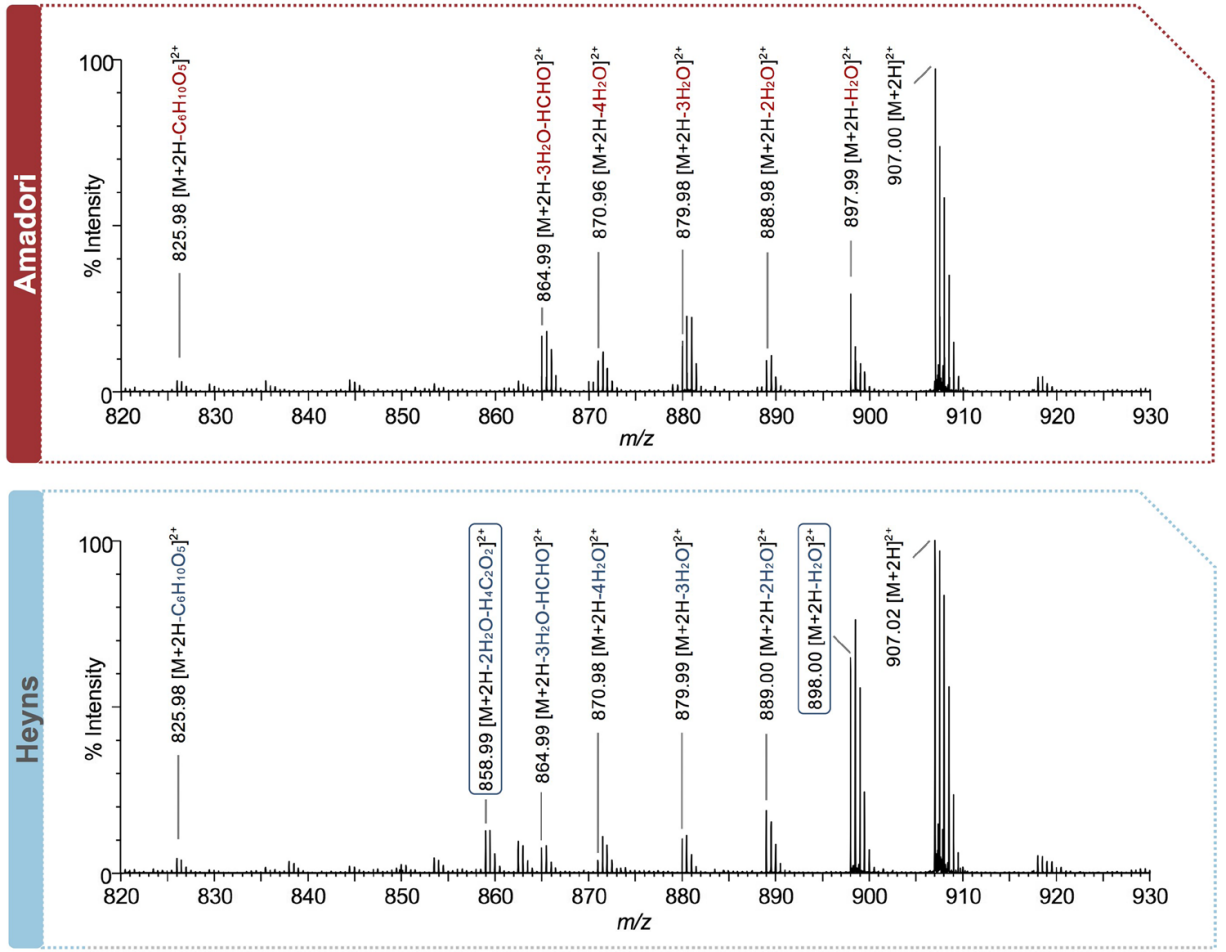
Tandem mass spectra of the doubly protonated quasimolecular ions detected at *m/z* 907.02 corresponding to fructosamine-modified (AEFAEVSK_Fru_LVTDLTK, Amadori product, top) and to glucosamine-modified peptides (**3a**, AEFAEVSK_Glc_LVTDLTK, Heyns product, bottom). The neutral losses characteristic for the sugar moieties are indicated above the peaks. Heyns-product fragment ions, which are exclusive (*m/z* 858.99) or significantly increased in their intensities (*m/z* 898.00) are highlighted with a frame. Data were acquired on an ESI-QTOF instrument using collision induced dissociation (CID) with an energy ramp from 18 to 50 V (32.1 V for *m/z* 907.02)

Fmoc-Lys(Glc,Boc)-OH (**2**) was used to synthesize the human serum albumin sequence AEFAEVSK_Glc_LVTDLTK (**3a**) (Table 1), which is a glucation site proposed as biomarker of type 2 diabetes (Frolov et al. 2014). A quantitative coupling of the glycated amino acid (**2**) was achieved in SPPS, when Fmoc-Lys(Glc,Boc)-OH was added in twofold molar excess, as indicated by the absence of the acetylated truncated peptide (Fig. 5). The fructated (**3a**) and the unmodified peptides (**3b**) synthesized in parallel were detected in the crude products with peak areas of 55% (Fig. 5) and 65% (data not shown), respectively, based on RP-HPLC (Table 1). The sequences were confirmed by the b- and y-ion series displayed in the tandem mass spectra acquired on a MALDI-TOF/TOF-MS (data not shown). Importantly, the unmodified sequence was not detected in the crude glycated product. However, a major byproduct (18%) was detected at 22.5 min with a mass shift of 26 *m/z*-units at Lys8 compared to **3a** or 188 *m/z*-units compared to **3b** (Fig. 5). The tandem mass spectra of this byproduct did not display the typical neutral loss pattern of glycated peptides (data not shown) indicating a modification on a hydroxyl group. Most likely the anomeric sugar hydroxyl group reacted with the Boc-group during acidic cleavage triggering an intramolecular cyclization yielding a 2-oxazolidinon derivative not prone to water losses in MALDI-MS/MS due to the stabilized ring structure (Supplement, Fig. S14). A similar byproduct was obtained at the amino acid level, when acidic treatment of Fmoc-Lys(Glc,Boc)-OH yielded a minor byproduct (Fig. 4). This proposed mechanism would be similar to the reaction of triphosgene or carbon dioxide (Niemi et al. 2018), when a cyclic carbamate modification is purposely introduced on Amadori products (Gallas et al. 2012) or oligosaccharides (Whitehead et al. 2017) enabling the synthesis of complex glycoconjugates. Although the presumed carbamate byproduct was well separated on RP-HPLC, its formation could be prevented for example by peracetylation of the sugar moiety. Nevertheless, such an approach would require an additional deprotection step reducing also the peptide yield (Tavernaro et al. 2015). The need for a separate carbohydrate deprotection can be eliminated by protecting hydroxyl groups as acid-labile acetals or 4-methoxybenzyl ethers, which are cleaved simultaneously with TFA-catalyzed release from solid support. The glycated (**3a**, Fig. 5) and unmodified peptides (**3b**, Supplement Fig. S16) were obtained in similar yields of 35%–40% and high purities (~ 96%). The structures were verified by NMR confirming the successful incorporation of glucosyllysine while excluding the mannosyllysine form (Figs. 4, S15, S17). Chemical shifts of the anomeric proton signals of the glucosyllysine derivative (**1a**) are in agreement with those detected for the glycated peptide (**3a**) indicating that the α-glucopyranosyl- and the β-glucopyranosl-forms are present at a 4:1 ratio. The tandem mass spectra of glucated (synthesized as described by (Spiller et al. 2017)) and fructated AEFAEVSK_Fru/Glc_LVTDLTK (**3a**) were dominated by characteristic neutral loss patterns (Fig. 6), i.e., consecutive losses of up to three water molecules (18 Da, 36 Da, and 54 Da) and a loss of three water and one formaldehyde molecules (84 Da). The Heyns product **3a** displayed an additional loss corresponding to 96 Da (*m/z* 858.99), which we reported earlier as specific for ketohexose-derived compounds (Frolov et al. 2006a; Corzo-Martínez et al. 2009).

**Table 1:** Yields, purities and monoisotopic masses of synthesized peptides

Sequence	Purity Crude [%]	Yield pure [%]	Purity purified [%]	Monoisotopic mass [Da]	
				Calc	Meas
AEFAEVSK_Glc_LVTDLTK (**3a**)	55	40	96	1811.9	1811.6
AEFAEVSKLVTDLTK (**3b**)	65	35	96	1649.9	1649.7

## Conclusions

The synthesis of a fructose-modified lysine derivative in two steps and its application as a building block for solid-phase peptide synthesis allowed the fast production of a Heyns peptide in high purity and good yield. This protocol overcomes the currently applied post-synthetic on-resin glycation of partially deprotected peptides. Despite using an unprotected sugar moiety, no epimerization was observed in the purified peptide. Only one major byproduct was formed, which was well separated by RP-HPLC, presumably due to incomplete cleavage of the Boc-group.

The low yield of the synthesis of Fmoc-Lys(Glc,Boc)-OH was partially attributed to the purification by RP-HPLC in the presence of 0.1% TFA. Better yields might be observed by ethyl acetate extraction to remove DMF and excess Boc_2_O (Stefanowicz et al. 2010). The extraction may even prevent the hydrolysis of the presumed acid-labile Fmoc-Lys(Man,Boc)-OH. However, the demonstrated chemoselective hydrolysis of epimeric Fmoc-Lys(Glc/Man, Boc) with subsequent purification by RP-HPLC might provide pure mannosyllysine in good yields. The presented synthetic strategy gives access to well-defined Heyns compounds of high purity, which will allow studying fructation and evaluating their potential to form toxic AGEs in more detail.

## Supplementary Information

Below is the link to the electronic supplementary material.

Supplementary file 1 (DOCX 1623 KB)
