# Supplementary material for: Solid-phase synthesis of d-fructose-derived Heyns peptides utilizing Nα-Fmoc-Lysin[Nε-(2-deoxy-d-glucos-2-yl),Nε-Boc]-OH as building block
Source: Amino Acids. 2021 May 2;53(6):881–91. doi: 10.1007/s00726-021-02989-7 (PMC8172402; doi:10.1007/s00726-021-02989-7)
Supplement: Supplementary file 1 — (DOCX 1623 KB) [file 726_2021_2989_MOESM1_ESM.docx]

**Solid phase synthesis of d-fructose-derived Heyns peptides utilizing N^α^-Fmoc-Lysin[N^ε^-(2-deoxy-d-glucos-2-yl),N^ε^-Boc]-OH as building block**

Sebastian Schmutzler^1,2^, Daniel Knappe^1,2^, Andreas Marx^3^, and Ralf Hoffmann^1,2^

^1^ Institut für Bioanalytische Chemie, Fakultät für Chemie und Mineralogie, Universität Leipzig, Leipzig, Germany

^2^ Biotechnologisch-Biomedizinisches Zentrum, Universität Leipzig, Leipzig, Germany

^3^ Site Management - Analytics, Merck KGaA, Darmstadt, Germany

* Corresponding authors

Prof. Dr. Ralf Hoffmann

Institut für Bioanalytische Chemie

Biotechnologisch-Biomedizinisches Zentrum

Deutscher Platz 5, 04103 Leipzig, Germany

E-Mail: bioanaly@rz.uni-leipzig.de

**Fig. S1** Chromatogram of crude N^α^-Fmoc-Lys[N^ε^-(2-deoxy-d-glucos/mannos-2-yl)]-OH (**1**) and mass spectra of the three most intense signals. The crude product was analyzed by RP-HPLC using a linear 30-min gradient from 3% to 57% aqueous acetonitrile containing 0.1% formic acid (absorbance recorded at 214 nm). The mass spectrum was recorded online on an ESI-iontrap-MS in positive ion mode. Charge states of the quasimolecular ions were calculated from the isotope patterns.

**Fig. S2** MALDI mass spectrum (top, *m/z* 300-650) and tandem mass spectra (bottom, collision energy of 1 kV, precursor: *m/z* 531.3, *m/z* 300-650) of crude product **1**. The assigned pyrylium and furylium ion structures due to consecutive neutral losses are characteristic for fructose-modified analytes.

**Fig. S3** Chromatogram and the ESI-MS (insert) of purified N^α^-Fmoc-Lys[N^ε^-(2-deoxy-d-glucos*-*2-yl)]-OH (**1a**). The compound was analyzed by RP-HPLC using a linear 30-min gradient from 3% to 57% aqueous acetonitrile containing 0.1% formic acid (absorbance recorded at 214 nm). The mass spectrum was recorded online on an ESI-iontrap-MS in positive ion mode from *m/z* 200 to 800. The small insert shows the isotope pattern of the protonated quasimolecular ion.


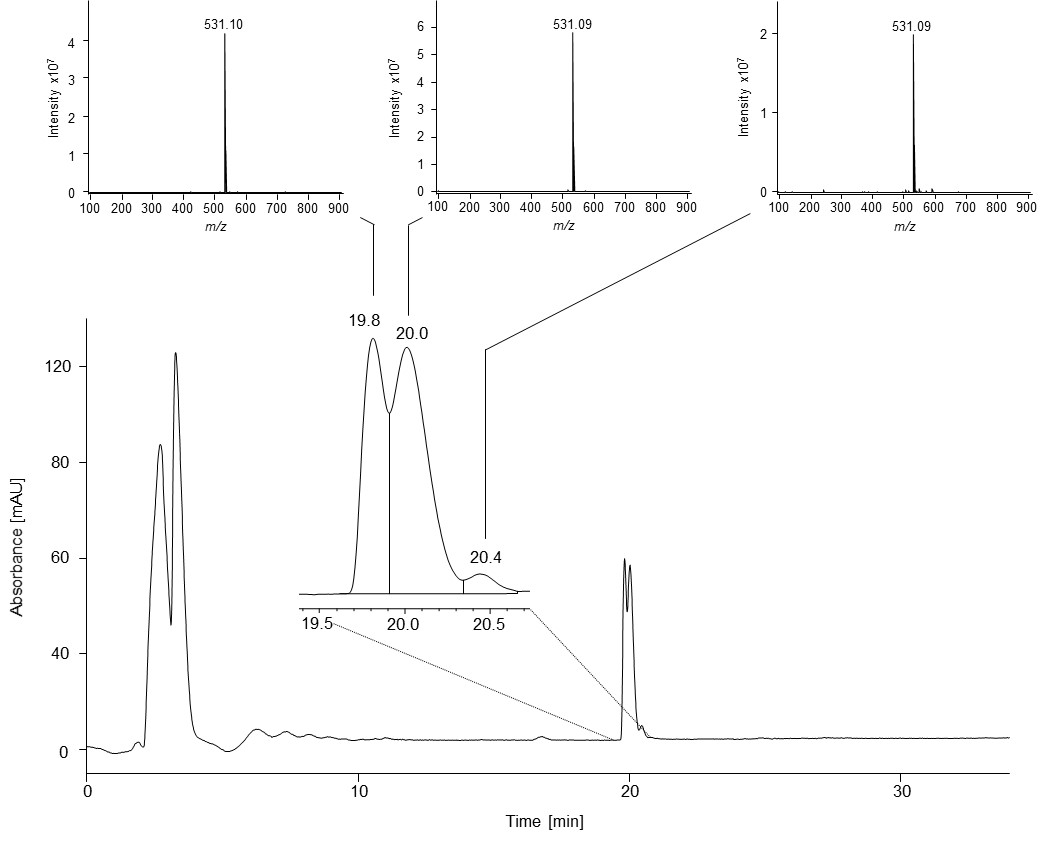


**Fig. S4** RP-Chromatogram of purified N^α^-Fmoc-Lys[N^ε^-(2-deoxy-d-glucos/mannos-2-yl)]-OH (**1**) in order to obtain the mannosyl compound. RP-HPLC used a linear 30-min gradient from 3% to 57% aqueous acetonitrile containing 0.1% formic acid (absorbance recorded at 214 nm). The mass spectrum was recorded online on an ESI-iontrap-MS in positive ion mode. Charge states of the quasimolecular ions were calculated from the isotope patterns.


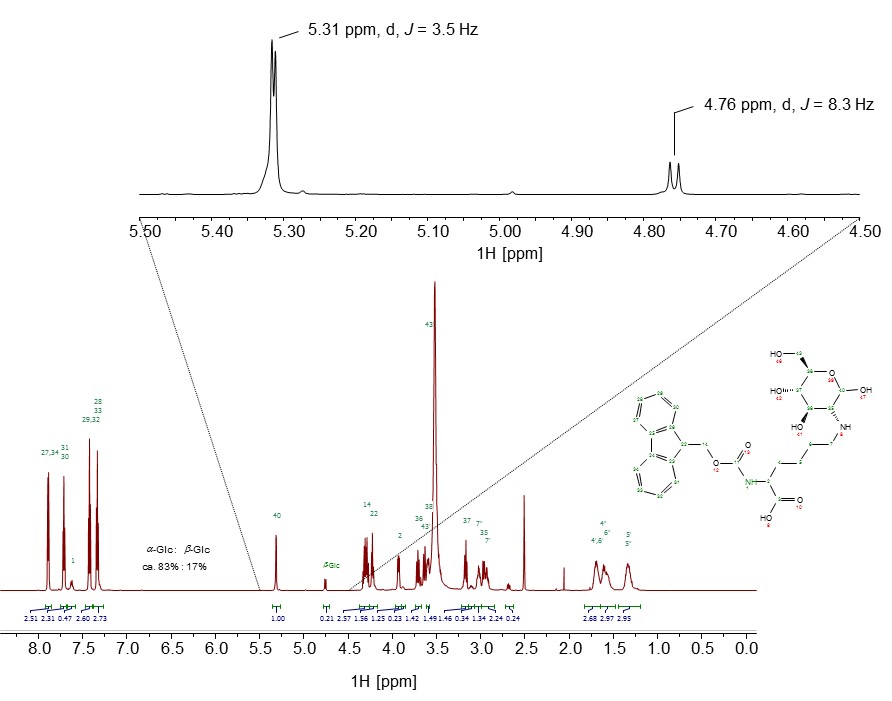


**Fig. S5** ^1^H-NMR spectrum of purified N^α^-Fmoc-Lys[N^ε^-(2-deoxy-d-glucos-2-yl)]-OH (**1a**) dissolved in DMSO-*d*_6_/D_2_O (700 MHz). The zoomed section (4.5-5.5 ppm) displays the chemical shifts and coupling constants of protons at C_1_’.

**
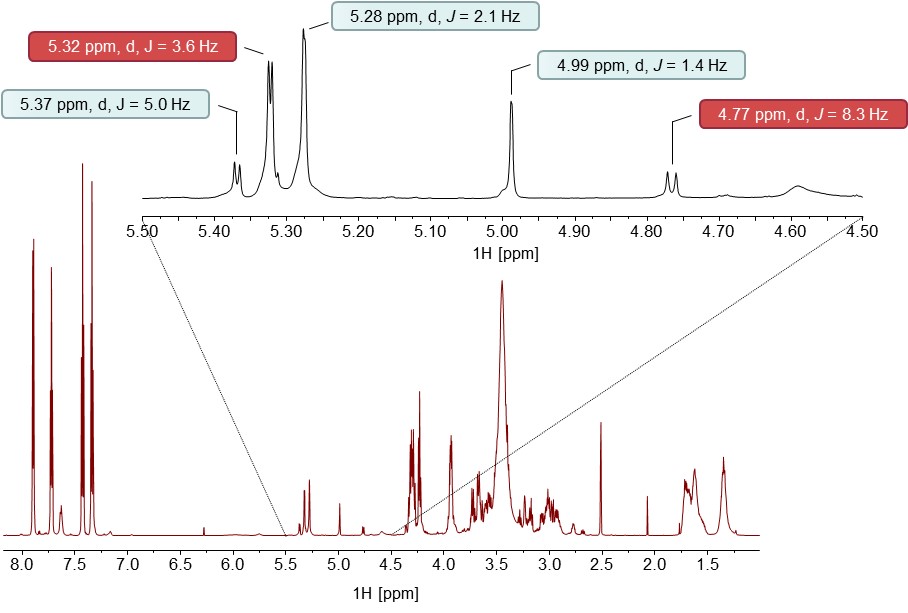
Fig. S6** ^1^H-NMR spectrum of purified N^α^-Fmoc-Lys[N^ε^-(2-deoxy-d-glucos/mannos-2-yl)]-OH (**1**) dissolved in DMSO-*d*_6_/D_2_O (700 MHz). The zoomed section (4.5-5.5 ppm) displays the chemical shifts and coupling constants of protons at C_1_’. Glucosyl- and mannosyl-derived signals are highlighted in red and blue, respectively.

**
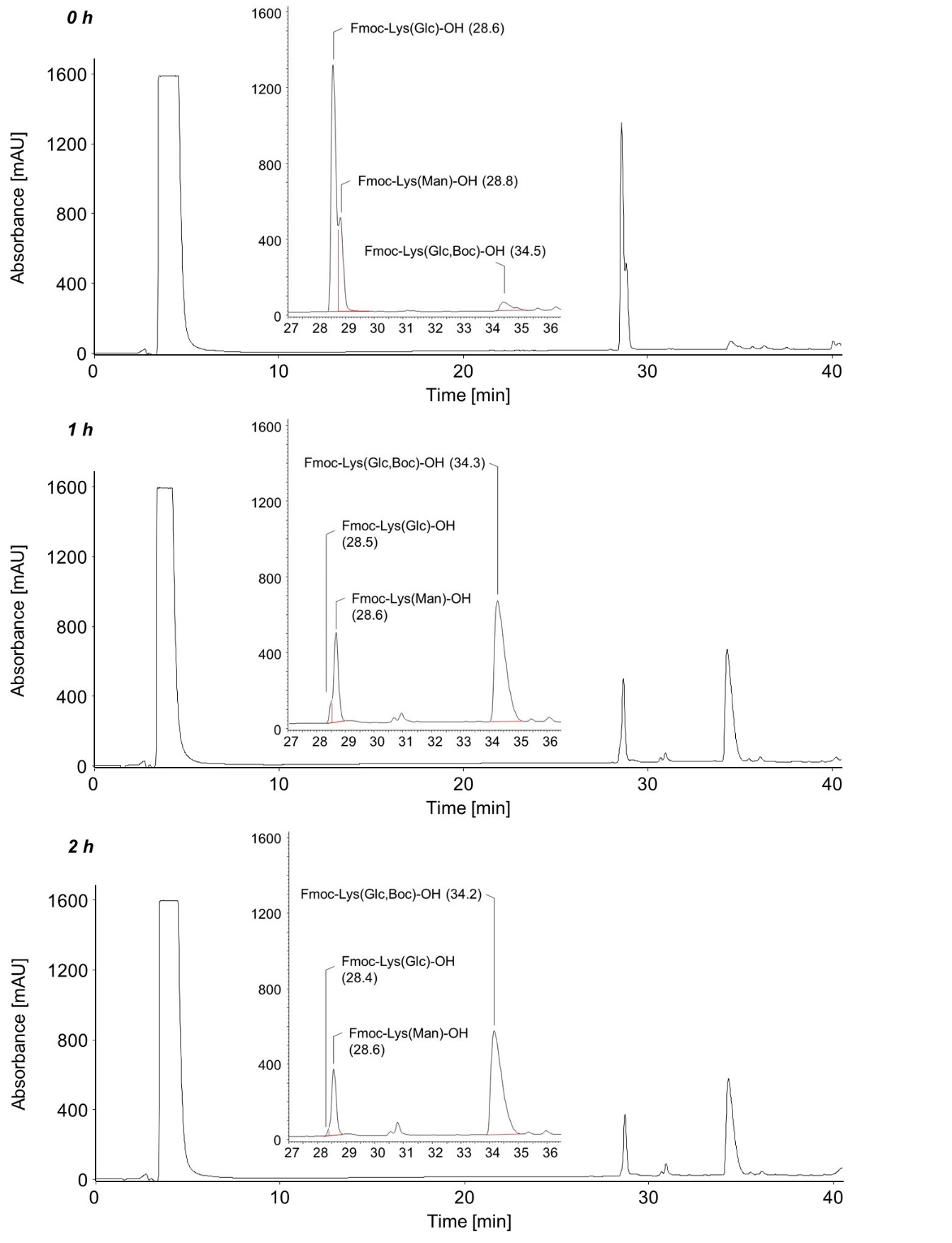
**

**Fig. S7** RP-chromatograms monitoring the Boc-protection of N^α^-Fmoc-Lys[N^ε^-(2-deoxy-d-glucos/mannos-2-yl)]-OH (**1**) after 0 h (top), 1 h (middle), and 2 h (bottom). RP-HPLC relied on a linear 30-min gradient from 3% to 57% aqueous acetonitrile containing 0.1% TFA (absorbance recorded at 214 nm). The small inserts show the chromatograms from 27 min to 36 min.

**Fig. S8** RP-chromatograms monitoring the Boc-protection of N^α^-Fmoc-Lys[N^ε^-(2-deoxy-d-glucos/mannos-2-yl)]-OH (**1**) after 0 h (top), 1 h (middle), and 1.5 h (bottom). RP-HPLC relied on a linear 30-min gradient from 3% to 57% aqueous acetonitrile containing 0.1% formic acid (absorbance recorded at 214 nm).


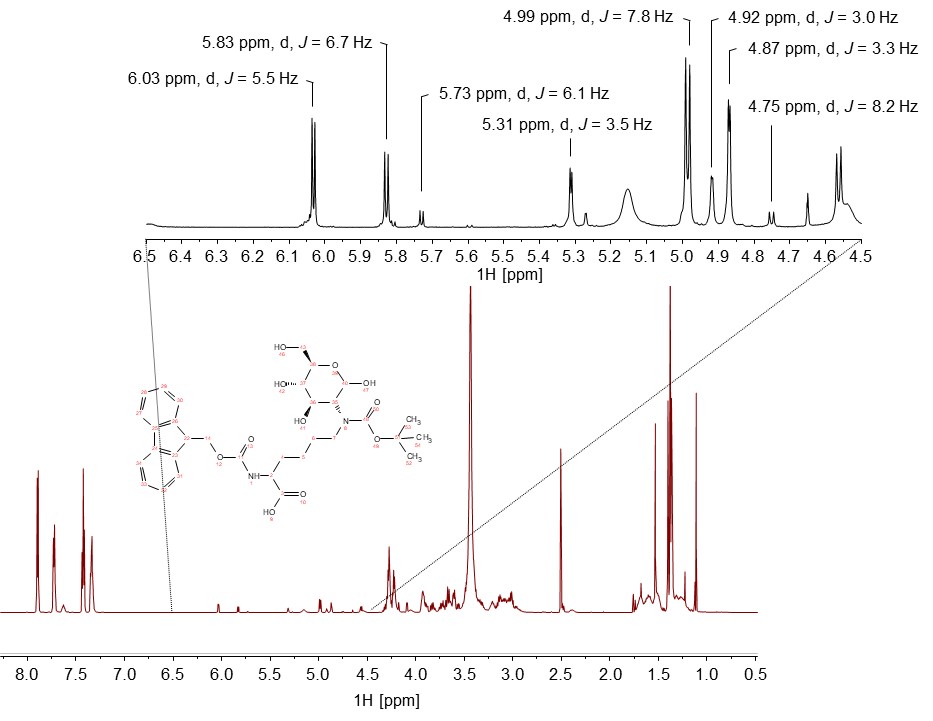


**Fig. S9** ^1^H-NMR spectrum of purified N^α^-Fmoc-Lys[N^ε^-(2-deoxy-d-glucos-2-yl,N^ε^-Boc)]-OH (**2**) dissolved in DMSO-*d*_6_/D_2_O (700 MHz). The zoomed section (4.5-6.5 ppm) displays chemical shifts and coupling constants of protons at C_1_’.

**
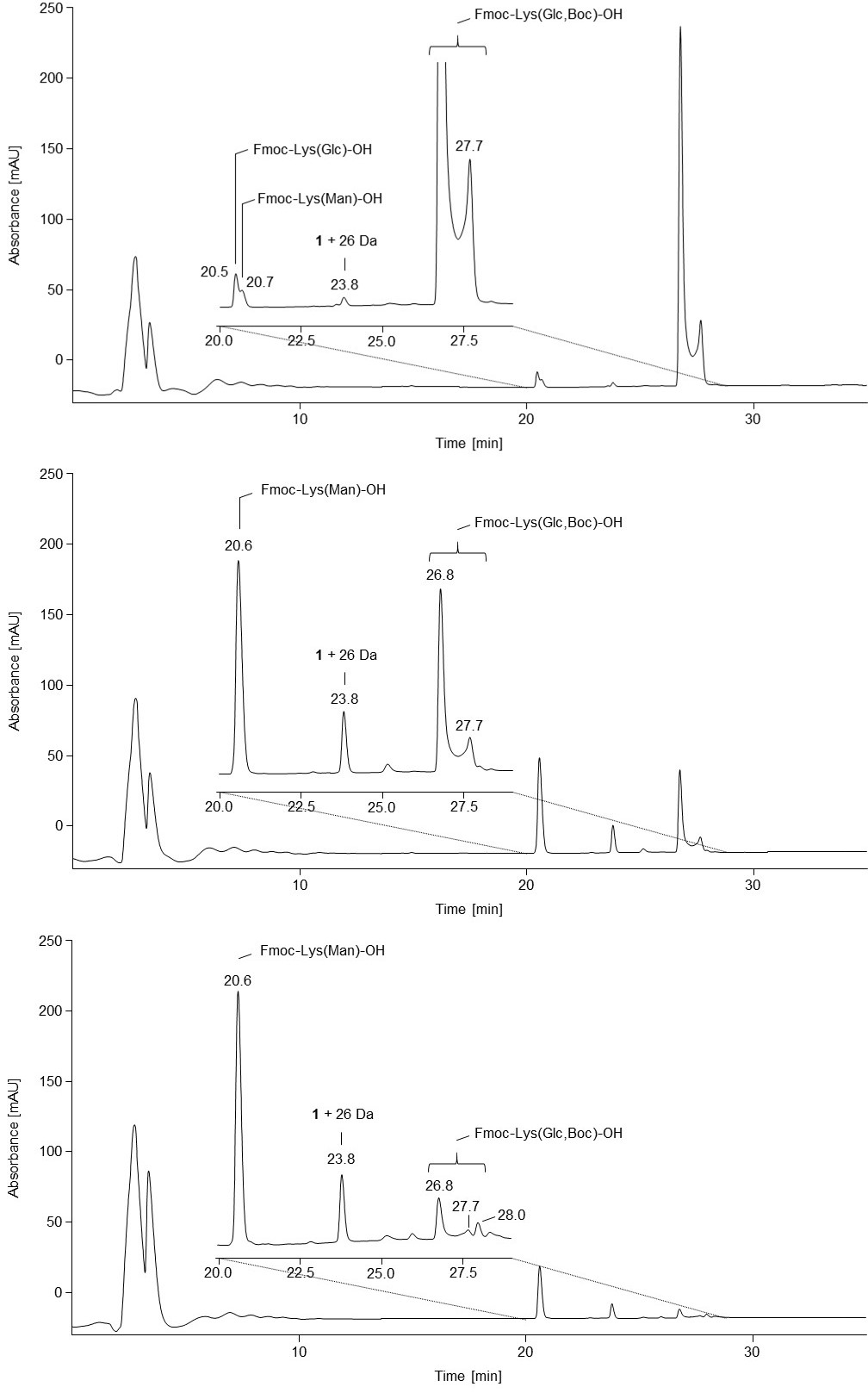
**

**Fig. S10** Chromatograms of N^α^-Fmoc-Lys[N^ε^-(2-deoxy-d-glucos-2-yl),N^ε^-Boc]-OH (**2**, top) purified by preparative RP-HPLC and later eluting byproducts (middle, bottom). RP-HPLC used a linear 30-min gradient from 3% to 57% aqueous acetonitrile containing 0.1% formic acid (absorbance recorded at 214 nm).

**Fig. S11** RP-Chromatogram of purified N^α^-Fmoc-Lys[N^ε^-(2-deoxy-d-mannos-2-yl)]-OH (**1b**) fractionated after Boc protection of compound **1**. RP-HPLC relied on a linear 30-min gradient from 3% to 57% aqueous acetonitrile containing 0.1% formic acid (absorbance recorded at 214 nm). The mass spectrum was recorded online on an ESI-iontrap-MS in negative ion mode from *m/z* 200 to 800. The small insert shows the isotope pattern of the deprotonated quasimolecular ion.


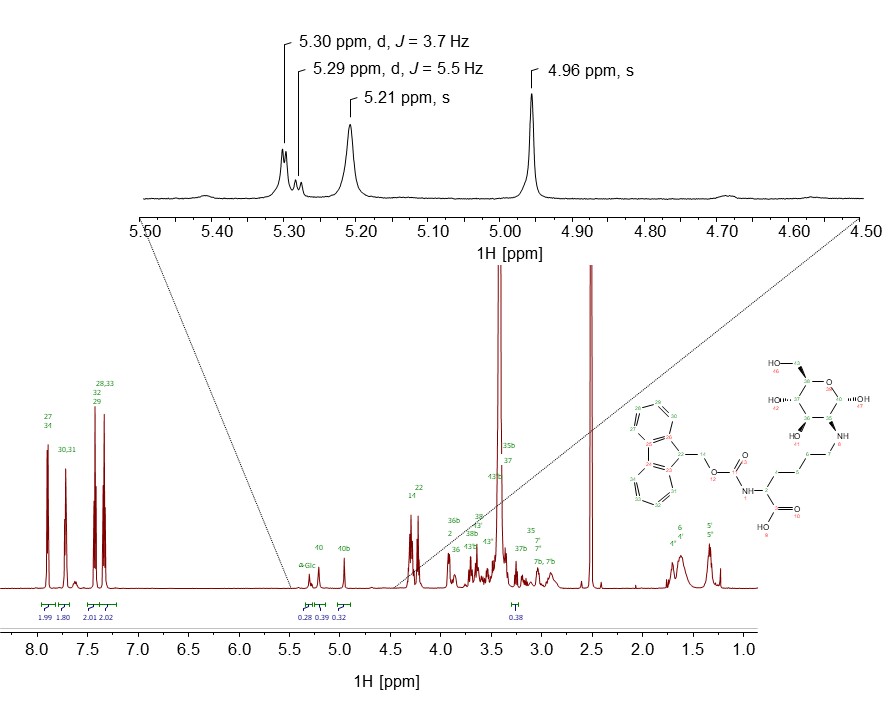


**Fig. S12** ^1^H-NMR spectrum of purified N^α^-Fmoc-Lys[N^ε^-(2-deoxy-d-mannos-2-yl)]-OH (**1b**) dissolved in DMSO-*d*_6_/D_2_O (700 MHz). The zoomed section (4.5-5.5 ppm) displays chemical shifts and coupling constants of protons at C_1_’.

**Fig. S13** Mass spectra of crude AEFAEVSK_Glc_LVTDLTK (**3a)** recorded in positive ion mode from *m/z* 500 to 1000 eluting from 20.4 min to 21.1 min (top, target compound) and at 22.5 min (bottom, byproduct). The small inserts show the isotope patterns of triply and doubly protonated quasimolecular ions. For further details see *Experimental*.

**Fig. S14** Reaction mechanism proposed for the formation of a byproduct observed in the synthesis of peptide AEFAEVSK_Glc_LVTDLTK (**3a**). The Boc-cleavage mechanism is adapted from Wang et. al (Wang J, Liang YL, Qu J (2009) Boiling water-catalyzed neutral and selective N-Boc deprotection. Chem Commun 5144–5146. https://doi.org/10.1039/b910239f).

**Fig. S15** ^1^H-NMR spectrum of purified peptide AEFAEVSK_Glc_LVTDLTK (**3a**) dissolved in DMSO-*d*_6_/D_2_O (700 MHz).


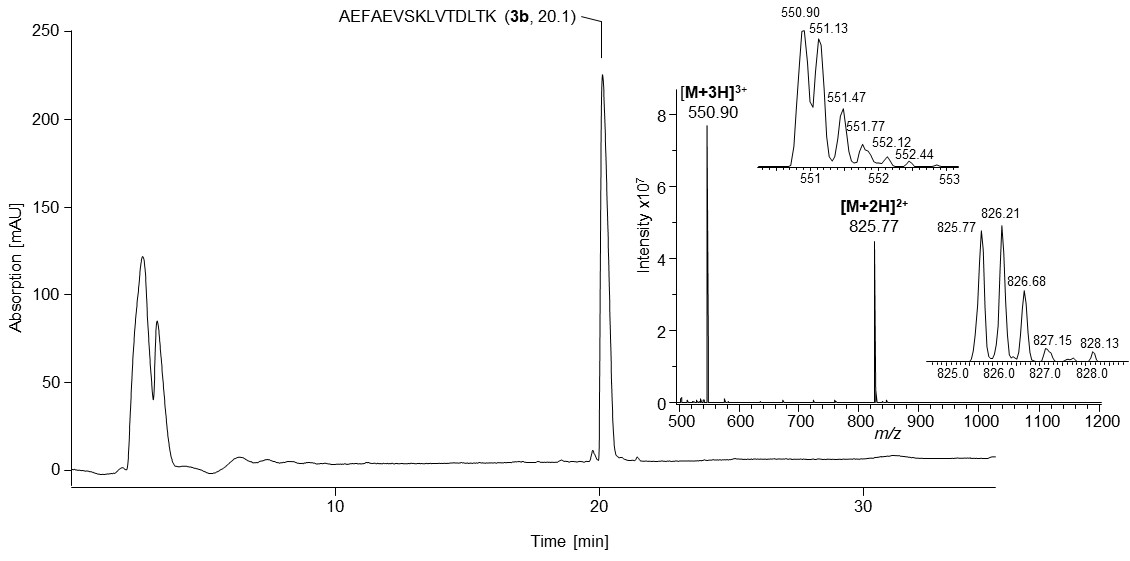


**Fig. S16** Chromatogram of purified peptide AEFAEVSKLVTDLTK (**3b**) and mass spectrum of the most intense peak. The peptide was analyzed by RP-HPLC using a linear 30-min gradient from 3% to 57% aqueous acetonitrile containing 0.1% formic acid (absorbance recorded at 214 nm). The mass spectrum was recorded online on an ESI-iontrap-MS in positive ion mode. The small inserts show the isotope patterns of the triply and doubly protonated quasimolecular ions at *m/z* 550.90 and *m/z* 825.77, respectively.

**Fig. S17** ^1^H-NMR spectrum of pure peptide AEFAEVSKLVTDLTK (**3b**) dissolved in DMSO-*d*_6_/D_2_O (700 MHz).


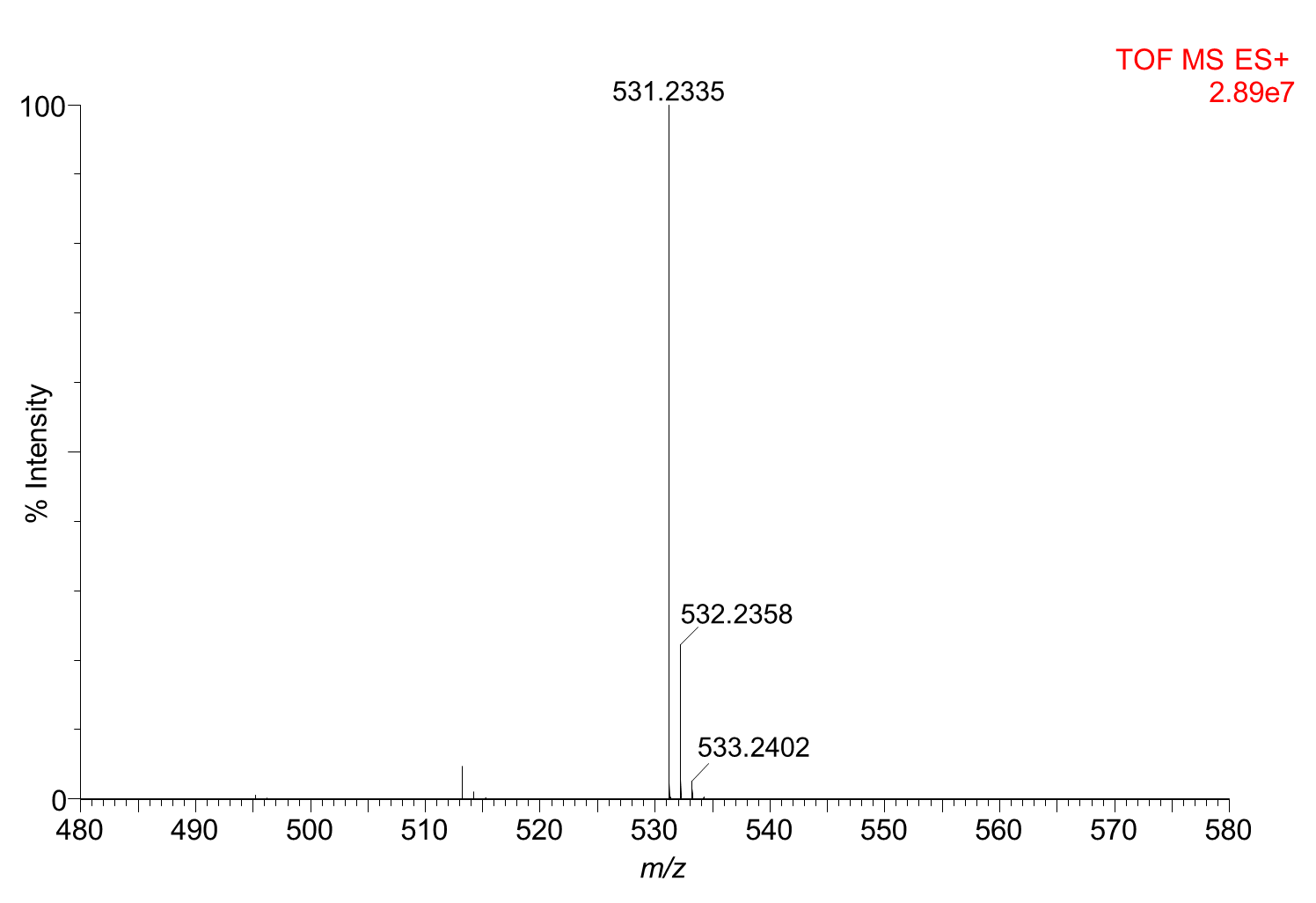


**Fig. S18** High-resolution mass spectra of purified N^α^-Fmoc-Lys[N^ε^-(2-deoxy-d-glucos/mannos*-*2-yl)]-OH (**1**) recorded in positive ion mode on an ESI-QTOF instrument using GluFib lock mass correction.


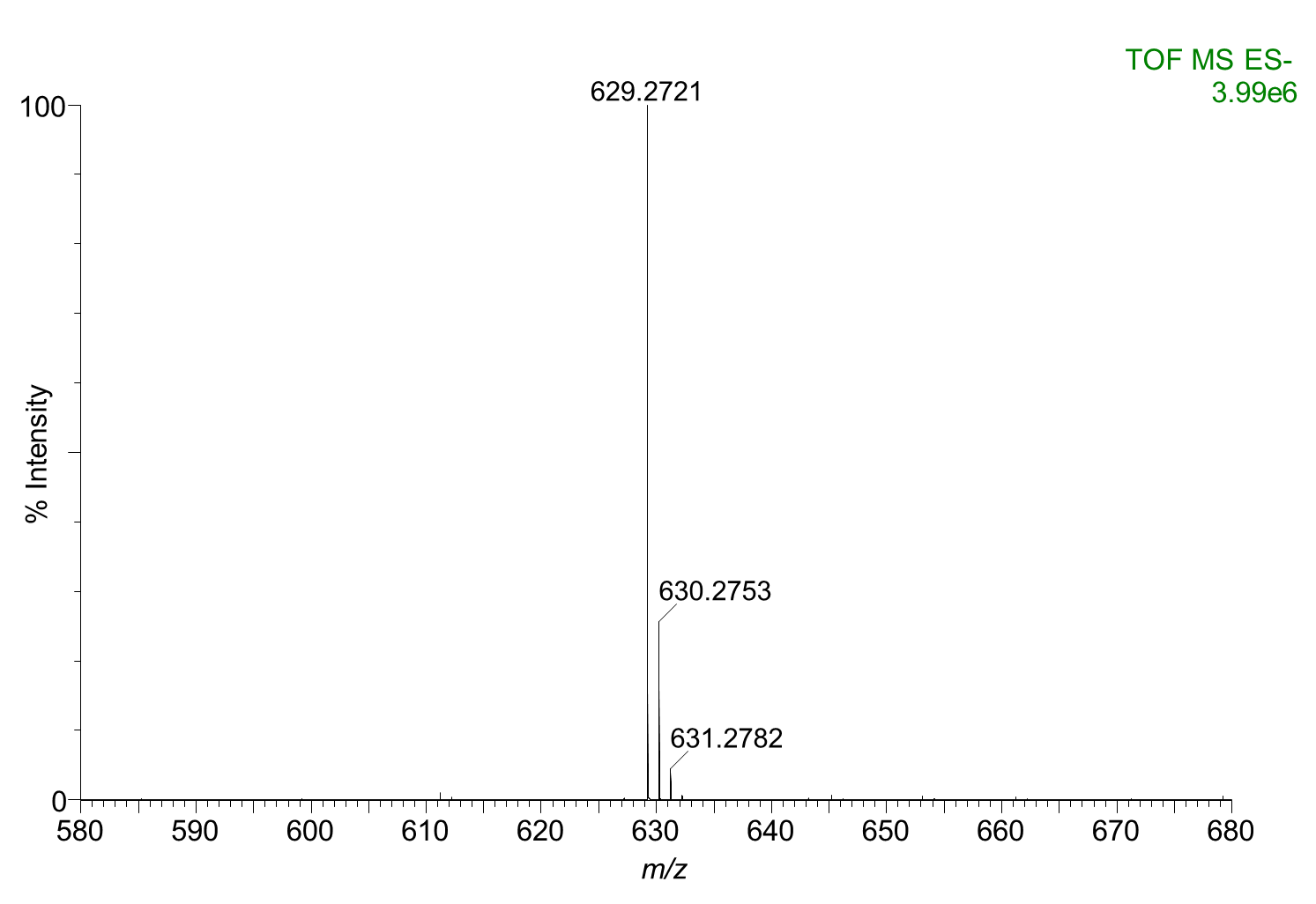
 **Fig. S19** High-resolution mass spectra of purified N^α^-Fmoc-Lys[N^ε^-(2-deoxy-d-glucos-2-yl),N^ε^-Boc]-OH) (**2**) recorded in negative ion mode on an ESI-QTOF instrument using GluFib lock mass correction.
